# Thank you to *The Lancet Regional Health - Europe*'s clinical and statistical peer reviewers in 2022

**DOI:** 10.1016/j.lanepe.2023.100590

**Published:** 2023-02-01

In this issue, the whole team at *The Lancet Regional Health – Europe* extend our sincere gratitude to all 805 statistical and peer reviewers who submitted a peer review report for the journal in 2022. Your contribution is one of the pillars of strength of the journal and we thank you for your critical and constructive feedback that has improved and refined the papers published in 2022. We look forward to continued collaboration in 2023 and beyond.

Cristiana Abbafati

Michael Absoud

Laith Abu-Raddad

Cornelia Adlhoch

Jan Egil Afset

Charles Agyemang

Sairah Ahmed

Zoe Aitken

Tatsuo Akechi

Alexandros Alexiou

Karel Allegaert

Frederick Altice

Yuri Amirkhanian

Zorana Andersen

Robert Anderson

Gerhard Andersson

Evangelos Th Andreakos

Kristin Andrejko

Melissa Andrew

Satinder Aneja

Laura Anselmi

Raffaele Antonelli-Incalzi

Ronen Arbel

Marc Arbyn

Richard Armitage

David Armstrong

Melina Arnold

Miqdad Asaria

Diane Ashiru-Oredope

Euan Ashley

Gro Askgaard

Johan Askling

Sachin Atre

Nathalie Auger

Philippe Autier

Li-Yuan Bai

Shasha Bai

Jennifer Baker

Marcel Ballin

Pascal Barbry

Enis Baris

Tony Barnett

Jenny Barrett

Leonardo Basco

Ugofilippo Basellini

David Baud

Markus Bauswein

Sina Bavari

Benjamin Bavinton

Eliza Beal

Thilo Beck

Luís Beck-da-Silva

Alexandre Belot

James Bentham

Marie Berg

Selina Berg

Joana Berger-Estilita

Johannes Berkhof

Matthias Bethge

Erwin Birnie

Carlo Biz

Andrea Bonetti

Louis Bont

Matteo Bonzini

Signe Borgquist

Rohan Borschmann

Yap Boum II

Anders Boyd

Rosemary Boyton

Robert Brackbill

Hannah Bradby

Paula Braitstein

Carol Brayne

Emilio Bria

Melanie Brinkmann

James Brophy

Felicity Brown

Raffaele Bugiardini

Danilo Buonsenso

Richard Thomas Burnett

Robyn Burton

Cristina Cadenas-Sanchez

Angelo Cagnacci

Miao Cai

Enrique Calderón

Johanna Callhoff

Peter M A Calverley

Cristina Calvo

Miguel Camafort Babkowski

Valentina Cambiano

Jackie Campbell

Marcello Candelli

Bin Cao

Rafael Cardoso

Ben Carter

Karen Casson

Marco Cavaleri

Elisabeth Celius

Edina Cenko

Keith Chappell

Esmita Charani

Yanin Chávarri-Guerra

Hiam Chemaitelly

Bin Chen

Li-Chia Chen

Marcus Chen

Jason Pui Yin Cheung

Wisit Cheungpasitporn

R Matthew Chico

Catherine Ruth Chittleborough

Eric Chow

Jane Chudleigh

Sumeet Chugh

Sheng-Chia Chung

Giancarlo Ciarelli

Catia Cilloniz

Hedi Claahsen - van der Grinten

Laura Coates

Leandro Dos Santos Coelho

Susan Coffin

John Coggon

Francesca Colavita

Vittoria Colizza

Intira Collins

Philippe Colson

Luke Connelly

Jennie Connor

Ana Maria Contardo Ayala

Andrew Copas

Rosie Cornish

Lara Coughlin

Benjamin Cowling

Patricia Coyle

Alison Crawshaw

Chiara cremolini

Julio Croda

Dan Crouse

Maggie Cruickshank

Andrea Dalbeni

Susanne Dalton

Clarissa Damaso

Andrei M Darie

Jayati Das-munshi

Matthias David

Grace Davie

Elisabetta De Cao

Alexandre de Figueiredo

Giovanni de Girolamo

Marlieke de Kraker

Pablo de Pedraza

Olivier de Schütter

Didier Debieuvre

Suzette Delaloge

Brendan Delaney

Lanfranco D'Elia

Rick Dersch

Don Des Jarlais

Mélanie Deschasaux-Tanguy

Delan Devakumar

Massimiliano Di Filippo

Antonio Di Sabatino

Joseph Dieleman

Olaf Dietrich

Linda Dirven

Stephanie Dixon

Therese Djärv

Charles R Doarn

Matthew Dodd

James Doidge

Pere Domingo

Jenny Downs

Jelena Drulović

Mignon du Plessis

William Dunning

Julien Edeline

Michael Edmonds

Anne Eggen

Eliudi Eliakimu

David Elliman

Sascha Ellington

Johan Engdahl

Armin Ensser

Friederike Erdmann

Christian Erikstrup

Janne Estill

Steen Ethelberg

Paolo Eusebi

Chiara Fabbri

Francesco Facchinetti

Christopher Fairley

Henrik Falhammar

Eric Faragher

Lilla Farkas

Rebecca Farrington

Forough Farrokhyar

Farshad Farzadfar

Michael Fehlings

Daniel Feikin

Jose Fernandez

Roshen Fernando

Clarissa Ferrari

Helen Fifer

Francis Finucane

Colin Fitzpatrick

Antoine Flahault

Roy Fleischmann

Hermes Florez

Barnaby Flower

Daniele Focosi

Francesco Fontana

Lisa Force

Nathan Ford

Anne-Marie Fors Connolly

Anna Forsberg

Yvonne Forsell

Carlos Magno Fortaleza

Clàudia Fortuny

Claire Foster

Monique Foster

Kathryn Foti

Maralyn Foureur

Frits M E Franssen

Anna Freni Sterrantino

Philippe Froguel

Gema Frühbeck

Elaine Fuertes

Petros Galanis

James Galloway

Silvano Gallus

Brenda Gannon

Lilia Ganova-Raeva

Camilo Garcia

Rodrigue Garcia

David García-Azorín

Lorra Garey

Mitchell Geffner

Mathieu Gendrot

Marco Geraci

Laurent Gerbaud

Jason Gill

Stuart Gilmour

Pere Ginès

Elisa Giovannetti

Nicolas Girard

Christian Georg Giske

Aharona Glatman-Freedman

Massimiliano Gnecchi

Daniel Golparian

Luis Goncalves

Ian Michael Goodyer

Herman Goossens

Kim Gradel

Giovanni Grasso

Michael Green

Christina Greenaway

Eve Griffin

Ulla Griffiths

Daniel Grint

Dan Grisaru

Martin Grobusch

Scott Grosse

Axel Grothey

Mariella Guerra

Freedom Gumedze

Sumit Gupta

Michelle Gurvitz

Mathias Haarhaus

Allan Hackshaw

Søren Hagstrøm

André Hajek

Wayne Hall

Ailish Hannigan

Peter Hansen

Tine Hansen

Katharine Harding

Iain Parry Hargreaves

Sally Hargreaves

Michael Harhay

Rowan Harwood

Cesare Hassan

Zaki Hassan-Smith

Aki Samuli Havulinna

Feng He

Yulei He

Christoph Heesen

Robert Heimer

Philip S Helliwell

Hartmut Hengel

Lena Henriksen

Marcus Herdener

Franz Hessel

Leonardo Heyerdahl

Benjamin Hibbert

Hirofumi Hirakawa

Roger Ho

Russell Hope

Daniel Horner

Susan Horton

Ahmed Hossain

Jessica Howell

Yunda Huang

Christian Hugo

Jacques Hugon

Benedikt Huttner

Benedikt Huttner

Agustin Ibanez

Armin Imhof

Leeberk Raja Inbaraj

Margaret Ip

Mary Ip

Sebastian Irudaya Rajan

Rodney Jackson

Lennart Jacobsson

Leila Janani

Meghna Jani

Asko Jarvinen

Domantas Jasilionis

Bjoern-Erik Jensen

Rachel Jewkes

Mark Jit

Christoffer Johansen

Christine Johnston

Jost Jonas

Linus Jonsson

Steven Julious

Amy C Justice

Orit Kaidar-Person

Sureshkumar Kamalakannan

Fatjona Kamberi

Günter Kampf

Mona Kanaan

Azza Karam

Marina Karanikolos

Beate Karges

Srinivasa Katikireddi

Scott Kehler

Mark Kelson

Zoe Kelson

Julia Kenny

Marcus Keogh-Brown

Robert Kerrison

Michael Keusgen

Sahil Khanna

Ali Khashan

Carolin Kilian

Joep Killestein

Jerome Kim

Lois Kim

Graham Kirkwood

Masafumi Kitakaze

Peter Klimek

Jonas Klingström

Gert Klug

Uwe Koedel

Evangelos Kontopantelis

Tulay Koru-Sengul

Karel Kostev

Joanne Kotsopoulos

Stefan Koudstaal

Georgia Kourlaba

Małgorzata Kowalska

Christian Kratz

Elizabeth Kristjansson

Maria Krutikov

Christine Kubiak

Irene Kuipers

Bernadette Kumar

Chirag Kumar

Yong-Fang Kuo

Hannah Kuper

Carlo La Vecchia

Sham Lal

Hiddo Lambers Heerspink

Joanne Langley

Anne Langsted

Iris Lansdorp-Vogelaar

Mette Bach Larsen

Eric Latimer

Eric Lau

Guy Launoy

Helen Lavretsky

Christopher Lavy

Andrew Laycock

Jose Luis Lazaro-Martinez

Jeffrey Lazarus

Scott Lear

Mariska Leeflang

Guillaume Lefèvre

Clara Lehmann

Jessica Leight

Katherine LeMasters

Isabelle Lemieux

Belinda Lennox

David Leon

Eyal Leshem

Michael Levin

Christelle Lévy

Dan Lewer

Gemma Lewis

Junhua Li

Linxin Li

Monica Sorina Licker

Nilton Lincopan

Per Eric Lindgren

Gregory Lip

Paul Lips

Mark Litaker

Jue Liu

Michael Livingston

Jonathan Livingstone-Banks

Caroline Lodge

Denis Logunov

Ellen Løkkegaard

Thomas Longden

Catherine Lord

Paul Loubet

Shun Lu

Expedito Luna

Ingeborg Lund

Jens Lundgren

Jay Lusk

Elsebeth Lynge

Ronan Lyons

Georgios Lyratzopoulos

Andrew Maas

Andrew Macadam

Joreintje Mackenbach

Graeme MacLennan

Michael Mæng

Claudio Maffeis

Stefania Maggi

Dianna Magliano

Maimuna Majumder

Atul Malhotra

Mamas Mamas

Lionel Mandell

Mattias Mandorfer

Mohammad Ali Mansournia

Valentina Marchese

Andrea Mariano

Michael Marks

Adilson Marques

Katherine Marshall

Esteban Martinez

Pablo Martinez-Camblor

Federico Martinon-Torres

Aileen Marty

Ana Marusic

Alice Masillo

Victor Masip

Farrah Mateen

Nicholas Matheson

Francesco Mattace

Citra Nurfarah Mattar

Fiona Matthews

Barbara Maughan

Mohsen Mazidi

Noel McElvaney

Francisco Medrano

Kirsty Mehring-Le Doare

Jawahar Mehta

Maria Melchior

Ignacio Mena

Fiona Mensah

Andrew Menzies-Gow

Stewart Mercer

Uta Merle

Stefano Merler

Tony Merriman

Benjamin Meyer

Gesine Meyer-Rath

Adam Milam

Alex Miras

Oren Miron

Hannah Mitchell

Isao Miyairi

Mohamed Mohamed

Viswanathan Mohan

Amir Mohsenpour

Ali Mokdad

Erika Molteni

Denis Mongin

Scott Montgomery

Caroline Moore

Daniel Morales

Lina Morch

Pedro Moro

Jean Moubarac

Vedang Murthy

Anne Myhre

Pierre Nabeth

Nico Nagelkerke

Mohsen Naghavi

Iris Nagtegaal

K M Venkat Narayan

Francesco Negro

Mayssam Nehme

Aidan Neligan

Laura Nellums

Charles Alexander Nelson

James M Neuberger

Sharon Neufeld

Alfred Neugut

Kathleen Neuzil

John Newton

Edmond Ng

Wan-Fai Ng

Todd Nicholas

Stig Nielsen

Suzanne Nielsen

Elena Nikiphorou

John Norrie

Kate Northstone

Tommy Nyberg

Malin Nygren-Bonnier

Éamonn O’Moore

Daniel O'Connor

Safiyyah Okoye

Andrew Olagunju

Dominic Oliver

Catherine Ong

Kathleen O'Reilly

Alberto Ortiz

Marlies Ostermann

Jill Owczarzak

Deepak Padmanabhan

Enny Paixao

Joseph Palamar

Christopher Palmer

Kuan-Yu Pan

Moty Pansky

Alberto Papi

Evridiki Patelarou

Ian Pavord

Lillian Pazvakawambwa

Ewan Pearson

Ulrich Pecks

Alma Pedersen

María Jesús Pérez Elías

Bruce Perkins

Anders Perner

Leigh Perreault

Eskild Petersen

Jesper Petersson

Salvatore Petta

Philippe Pibarot

Michael Pillinger

Stefan Pilz

Rosa Pinto

Maryam Piram

Lionel Piroth

Prisco Piscitelli

Alexandra Pitman

Lyudmila Pivina

Mathias Pletz

Robert Podolsky

Marina Pollán

Wendy Post

Tonia Poteat

Julien Pottecher

Anton Pottegård

Kameshwar Prasad

Francesco Prattichizzo

Maria Prendecki

Robin Prescott

A Matthew Prina

Kathy Pritchard-Jones

Charlotte Probst

Romana Pylypchuk

Wendi Qian

Jennifer Quint

Angela Raffle

Kaja Rahu

Massimo Ralli

Pilar Ramon-Pardo

Jonas Ranstam

Jonas Ranstam

Otavio Ranzani

Prashant Rao

Jean Philippe Rasigade

Yury Razvodovsky

Eleanor Rees

Jürgen Rehm

Juergen Reichardt

Jaap C Reijneveld

Kyndaron Reinier

Ulrich Reininghaus

Giuseppe Remuzzi

Eldon Raj Rene

Christel Renoux

Ana Requena-Mendez

Chin Kook Rhee

Angela Dalia Ricci

Alan S Rigby

Guido Rindi

Marco Ripa

Scott Ritchie

Chris Robertson

Natalia Rodriguez

Carlos Rodriguez-Galindo

Jeanine Roeters van Lennep

Pierre Simon Rohrlich

Amanda Rojek

Casper Rokx

Benjamin Rolland

Andres Roman-Urrestarazu

Rodney Rosalia

Zeev Rosberger

Markus Rose

Rafael Rosell

Måns Rosén

Joseph Rossano

Peter Rossing

Christina Rostad

Ali Rostami

Ashok Roy

Alberto Ruano-Ravina

Dennis Rubbenstroth

Jon Rueda

Richard E K Russell

Eva Ruzic-Sabljic

Piotr Rzymski

Séverine Sabia

Caroline Sabin

Manish Sadarangani

Marc Saez

Joaquin Salas-Coronas

Paulo H N Saldiva

Dirk Sander

Francesco Sannino

Benn Sartorius

Janarthanan Sathananthan

Rob Saunders

Thomas Schaible

Peter Schielen

Maarten Schim van der Loeff

Berit Schiøttz-Christensen

Patricia Schlagenhauf

Kristan Schneider

Susanne Schneider

Ulf Schönermarck

Karen Schreiber

Philipp Schuetz

Grant Schulert

Andreas Schuppert

Robert Scragg

Petar Seferović

Sharvan Sehrawat

Yu M Semenova

Jan Semenza

Gianluca Serafini

Raphaele Seror

Tom Shakespeare

Anna Shalimova

Jonathan Shaw

Daisy Shepherd

Tinevimbo Shiri

Vladimir Shkolnikov

Anna Sick-Samuels

Alex Sigal

Sergio Silverio

Aymeric Silvin

Stuart L Simpson

Don Sin

Archana Singh-Manoux

Daisy Singla

Valérie Siroux

Rod Skinner

David Skuse

David Smadja

William Small Jr

Neil Smart

Mike K P So

Maria Socias

Anne Lee Solevåg

Sunil Solomon

Mary Beth Son

Gabe Sonke

Joan Soriano

Antoni Soriano-Arandes

Mark Speechley

Jane Speight

Matthew Spittal

Lisa Stamp

Milan Stanojevic

Susan Stark

Giulio Stefanini

Coen Stehouwer

Douglas Steinke

Jo Stenehjem

Terence Stephenson

Sharon Stevelink

Charles Stiller

James Stone

Pasquale Strazzullo

Franc Strle

Matej Stuhec

L Suzanne Suggs

Jennifer Sumner

Johanne Sundby

Alexander Supady

Susan Sutherland

Norbert Suttorp

Leslie Swartz

Erin Symonds

Casey Taft

Emanuela Taioli

Carlo Tascini

Harry Tattan-Birch

Scott Tebbutt

Anders Tegnell

Paula ter Wengel

Vincent Thibault

Thomas Thiele

Claudius Thomé

Reimar Thomsen

Marianne Thoresen

Yasuharu Tokuda

Andrew Tomkins

Cathryn Tonne

Steve Torr

Antoni Torres

Sergi Trias-Llimós

Adam Trickey

Timir Tripathi

Palak Trivedi

Hueijen Tsai

Georgios Tsivgoulis

Vivien Tsu

Damien Tully

Barbara J Turner

Alexandar Tzankov

Agne Ulyte

Brigid Unim

Benedetta Vai

Maritta Välimäki

Carissa van den Berk-Clark

Bert Jan H van den Born

Maria van den Muijsenbergh

Simone van der Sar

Veronika van der Wardt

Marieke J van der Werf

Luke van Rhoon

Zsuzsanna Varga

Maria Vehreschild

Bruno Vellas

Vicente Verez-Bencomo

Mette Vesterhus

Rute Vieira

Marianna Virtanen

Otto Visser

Michele Vitacca

Jasper Vleugels

Arndt Vogel

Arndt Vogel

Tamar Wainstock

Naomi Walker

Timothy Walsh

Jianwei Wang

Philip Ward

Heiner Wedemeyer

James Cheng-Chung Wei

Paul Weiss

Claire Welsh

Paul Welsh

Xue Wen

Lukas Weseslindtner

Virginie Westeel

Adam Wheatley

Dominic Wichmann

John Williams

Fabian Winiger

Michel Wolff

Brian Wong

Robert Wong

George Woody

Ian Woolley

Gillian Worthy

Olivier Wouters

Tomasz Wrob́el

Jianhua Wu

Jie Wu

Steven Wyatt

Huichun Xu

Prashant Yadav

Dafna Yahav

Bo-Yi Yang

Wan Yang

Andrew David Yeoman

Bernadette Young

Anna Yousaf

Ge Yu

Kun-Hsing Yu

Francesco Zaccardi

Gregory Zacharewicz

Laura Zambrano

Mariabeatrice Zazzara

Fengqing Zhang

Ping Zhang

Xu-Sheng Zhang

Fengmin Zhao

Feng-Cai Zhu

Yifan Zhu

Ariana Znaor

Ania Zylbersztejn

